# Unlocking the Promise of Antitumor Hyperthermia‐Immunotherapy with Spiky Surface Topology

**DOI:** 10.1002/advs.202415868

**Published:** 2025-02-18

**Authors:** Muyue Yang, Yan Yu, Tongxin Ge, Qiuyi Zhu, Ai Zhuang, Wenxing Wang, Xianqun Fan

**Affiliations:** ^1^ Department of Ophthalmology Shanghai Ninth People's Hospital Shanghai JiaoTong University School of Medicine Shanghai 200011 China; ^2^ State Key Laboratory of Eye Health Shanghai Jiao Tong University Shanghai 200011 China; ^3^ Shanghai Key Laboratory of Orbital Diseases and Ocular Oncology Shanghai 200011 China; ^4^ Department of Chemistry State Key Laboratory of Molecular Engineering of Polymers Laboratory of Advanced Materials Fudan University Shanghai 200433 China

**Keywords:** immunotherapy, magnetic hyperthermia, spiky particles, uveal melanoma

## Abstract

Uveal melanoma (UM) is the most prevalent primary intraocular malignant tumor in adults with high mortality rate. Recently, immunotherapy has shown great success in other tumors, however, its therapeutic effect in UM is unsatisfactory, possibly due to the insufficient immune cell infiltration and low immunogenicity of UM. Thus, an efficient therapeutic strategy to reverse the immunosuppressive tumor microenvironment is required. Herein, a PD‐L1 modified hierarchical structure consisting of a magnetic Fe_3_O_4_ core and spiky silica shell (MNP@Spiky/PD‐L1) is developed to reverse the immunosuppressive tumor microenvironment and trigger powerful antitumor immune responses. The MNP@Spiky can induce enhanced immunogenic cell death as well as physical activation of innate immunity. First, tumor cells are disrupted directly by magnetic hyperthermia effect and released tumor‐associated antigens to initiate anti‐tumor immune responses. Meanwhile, the spiky surface of MNP@Spiky augmented tumor antigen uptake as well as maturation of dendritic cells through inflammasome activation. By further associating with PD‐L1‐targeting antibody, MNP@Spiky/PD‐L1 reversed the immunosuppressive tumor microenvironment and triggered powerful antitumor immune responses. Overall, this synergistic therapeutic strategy effectively reprogramed tumor microenvironment and achieved tumor eradication, which sheds light on clinical UM immunotherapy.

## Graphical abstract

MNP@Spiky nanoparticles are designed for efficient uveal melanoma treatment. After intraocular injection, tumor cells are disrupted directly by magnetic hyperthermia effect caused by the Fe_3_O_4_ core and released tumor‐associated antigens to initiate anti‐tumor immune responses. The spiky surface promoted the maturation of dendritic cells through inflammasome activation. By further associating with PD‐L1, MNP@Spiky/PD‐L1 activated CTLs and further eradicated tumor cells.

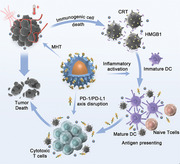

## 1 Introduction

Uveal melanoma (UM) is the most common intraocular malignancy in adults with high mortality rate.^[^ ^1^ ^]^ The prognosis for UM patients is very poor with 5‐year overall survival rate no more than 50%.^[^ ^2^, ^3^ ^]^ Conventional therapies including surgical resection, chemotherapy and radiotherapy have limited efficacy and often lead to severe side effects.^[^ ^4^, ^5^, ^6^ ^]^ In recent years, cancer immunotherapy by revitalizing exhausted cytotoxic T lymphocytes (CTLs), further motivating immune system to recognize and eradicate tumor cells, has gained significant momentum in clinical treatment, including immune checkpoint blockade (ICB), cancer vaccines, adoptive T cell therapy, and cytokine therapy.^[^ ^7^, ^8^ ^]^ Among them, ICB therapy, especially programmed cell death protein 1 (PD‐1)/programmed cell death ligand 1 (PD‐L1) axis regulation, has revolutionized traditional tumor therapy and achieved complete remission in multiple malignancies.^[^ ^9^, ^10^, ^11^ ^]^ Unfortunately, only a few patients suffering from UM have benefitted from ICB treatment.^[^ ^12^, ^13^ ^]^ One of the formidable challenges is that UM is a “cold” tumor with insufficient immune cell infiltration and low immunogenicity.^[^ ^14^, ^15^ ^]^ To date, various approaches have been harnessed to enhance its immunogenicity. One of the promising strategies is to trigger the immunogenic cell death (ICD) of tumor cells, which can release damage associated molecular patterns (DAMPs) and tumor‐associated antigens (TAAs), as immunostimulatory signals, thus eliciting antitumor immune responses.^[^ ^16^, ^17^, ^18^, ^19^ ^]^ Recently, magnetic hyperthermia therapy (MHT) with remote spatiotemporal controllability, desirable tissue penetration, and minimal damage to normal tissue has emerged as a future therapeutic modality for ICD induction.^[^ ^20^, ^21^, ^22^, ^23^, ^24^ ^]^ However, tumor antigens generated by ICD alone may not be enough for UM immunotherapy possibly due to the extremely low mutation burden of UM, which results in less production of neoantigens and a lower chance of immune recognition. Hence, it is imperative to exploit an approach to further enhance immunorecognition in UM immunotherapy.

**Scheme 1:**
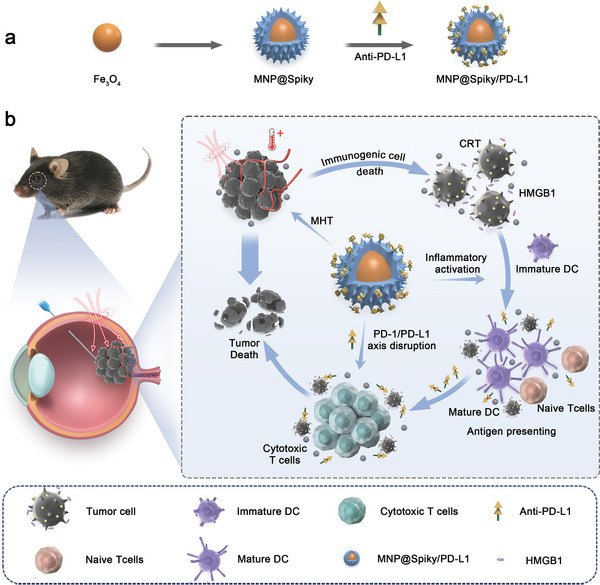
Schematic illustration of the construction of MNP@Spiky/PD‐L1 and its therapeutic effect on UM.

It is well known that the spiky‐like surface of multiple pathogens could facilitate their adhesion on the membrane of immune cells and elicit strong immune activation. Inspired by this, artificial nanopillar structures mimicking the surface of pathogens were designed to induce robust immune responses, and physical activation of innate immunity by spiky particles has been reported.^[^ ^25^, ^26^ ^]^ Herein, we propose to introduce physical activation of innate immunity to enhance MHT‐induced ICD in UM immunotherapy by fabricating magnetic Fe_3_O_4_ nanoparticles coated with spiky silica shell (MNP@Spiky) (**Scheme** **1**). In exposure to the high frequency alternating magnetic field (AMF), the Fe_3_O_4_ core exerted a magnetic hyperthermia effect to directly disrupt tumor cells and evoke abundant ICD, which promotes the recruitment and maturation of dendritic cells (DCs). Meanwhile, the spiky surface of MNP@Spiky facilitated its cellular uptake by DCs and augmented tumor antigen uptake as well as DC maturation through inflammasome activation. By further associating with PD‐L1‐targeting antibody, MNP@Spiky/PD‐L1 reversed the immunosuppressive tumor microenvironment and triggered powerful antitumor immune responses, generating excellent antitumor efficacy and preventing tumor recurrence. Taken together, this synergistic therapeutic strategy effectively reprogramed tumor microenvironment and achieved tumor eradication, which sheds light on clinical UM immunotherapy.

MNP@Spiky/PD‐L1 was composed of a magnetic Fe_3_O_4_ core, a spiky silica shell, and PD‐L1‐targeting antibody. After intraocular injection, tumor cells were disrupted directly by magnetic hyperthermia effect caused by the Fe_3_O_4_ core and released tumor‐associated antigens to initiate anti‐tumor immune responses. Additionally, the spiky surface of MNP@Spiky promoted tumor antigen uptake and maturation of dendritic cells through inflammasome activation. By further associating with PD‐L1, MNP@Spiky/PD‐L1 activated CTLs and further eradicated tumor cells.

## 2 Results and Discussion

### 2.1 Preparation and Characterization of MNP@Spiky

**Figure 1:**
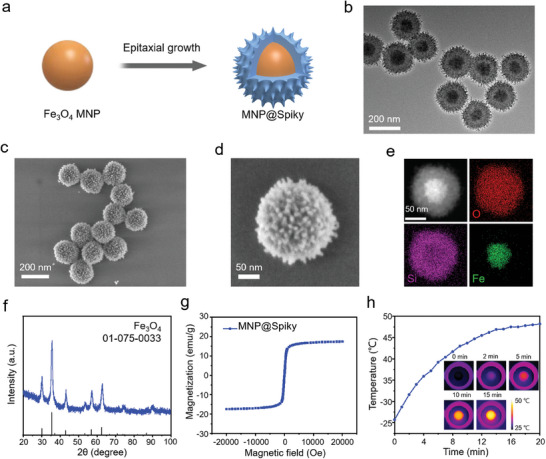
Structure and property characterization of MNP@Spiky. a) Schematic illustration of the synthesis of MNP@Spiky. b) TEM image of the obtained MNP@Spiky. c, d) SEM images of the obtained MNP@Spiky. e) Element mapping of the obtained MNP@Spiky. f) XRD spectrum of MNP@Spiky. g) The magnetization curve of MNP@Spiky. h) Heating profiles of MNP@Spiky: temperature–time curve and real time in vivo IR thermal images.

A schematic illustration of the fabrication of MNP@Spiky was presented in **Figure** **1**a. A single‐micelle epitaxial growth method was utilized to grow spiky silica shell on spherical Fe_3_O_4_ NPs for the preparation of MNP@Spiky.^[^ ^27^ ^]^ As shown in transmission electron microscopy (TEM) image (Figure 1b), MNP@Spiky showed a distinct core‐shell structure composed of a spherical magnetic nanoparticle with a diameter of≈80 nm and a spiky silica shell covered with vertical silica nanospikes (≈20 nm in length and≈7 nm in diameter). The hydrodynamic size of MNP@Spiky was measured to be 230 nm (Figure S1, Supporting Information). Scanning electron microscopy (SEM) images (Figure 1c,d) further revealed the unique spiky surface topology of the monodispersed MNP@Spiky. The distribution of different elements (Fe, Si, and O) in one MNP@Spiky identified by the energy dispersive spectrometer (EDS) mapping (Figure 1e) further demonstrated the distinctive core‐shell structure and the unique spiky surface. The X‐ray diffraction (XRD) pattern showed that the diffraction peaks of MNP@Spiky matched well with the Fe_3_O_4_ phase (JCPDS No. 01‐075‐0033, Figure 1f). The MNP@Spiky exhibited a good magnetic response with magnetization saturation at 16 emu/g (Figure 1g). Subsequently, the magnetic heating efficiency was evaluated under thermal imaging (Figure 1h) and the MNP@Spiky demonstrated stable magnetic‐thermal performance. The temperature of the MNP@Spiky aqueous solution increased from 26 to 48 °C after exposure to an AMF after 20 min. MNP nanoparticles with a smooth surface (MNP@Smooth) were synthesized by coating a smooth silica layer on the spherical Fe_3_O_4_ core to serve as the contrast sample (Figure S2‐4). All samples exhibited good stability in PBS solution (Figure S5–7).

### 2.2 MNP@Spiky‐Mediated MHT and ICD Cascade In Vitro

**Figure 2:**
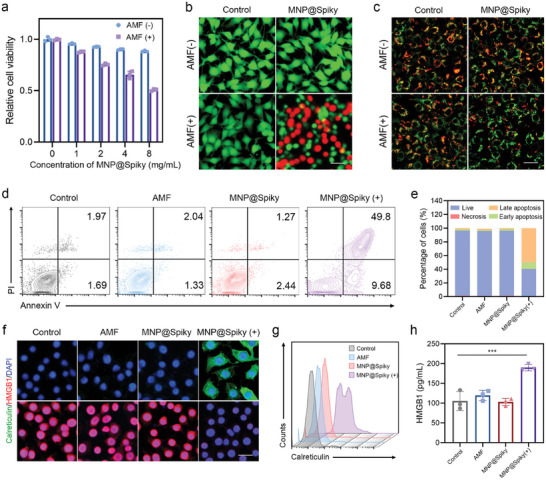
MNP@Spiky‐Mediated MHT and ICD Cascade In Vitro. a) Relative cell viability of B16F10 cells after treatment with different concentration of MNP@Spiky, followed with or without AMF. b) Live and dead assay of B16F10 cells with different treatments. Red fluorescence represents dead cells and green fluorescence represents live cells. Scale bar: 10 µm. c) JC‐1 staining assay of B16F10 cells. In normal cells, JC‐1 dye aggregates and emits red fluorescence, whereas JC‐1 monomers display as green fluorescence at low mitochondrial membrane potentials. Scale bar: 10 µm. d) Flow cytometry and e) the corresponding quantitative analysis of cell apoptosis in each group. f) Immunofluorescence staining showing CRT (green) exposure and HMGB1 (red) release in B16F10 cells with various treatments. Scale bar: 10 µm. g) Flow cytometry analysis of CRT exposure in B16F10 cells. h) The levels of HMGB1 in the supernatants of B16F10 cells. The statistical significance was calculated via Student's *t* test. Data are represented as the mean ± SD. ***p < 0.001.

To explore the biological function of MNP@Spiky, the interaction of MNP@Spiky with B16F10 cells was first investigated. Tumor cells were incubated with FITC‐labeled MNP@Spiky. Strong fluorescent signal was observed around the nuclei, revealing efficient cellular uptake of MNP@Spiky (Figures S8 and S9, Supporting Information). Then in vitro cytotoxicity assays were conducted. MNP@Spiky showed slight cytotoxicity to B16F10 cells and NIH/3T3 cells (normal fibroblasts), suggesting high biocompatibility of MNP@Spiky (**Figure** **2**a; Figures S10–12, Supporting Information). However, significant cytotoxicity was detected in the MNP@Spiky‐treated group under AMF, confirming its MHT effect. As expected, B16F10 cells were killed in a dose‐dependent manner upon exposure to AMF, and the cell viability was 50.8% at a concentration of 8 mg mL^−1^ (Figure 2a). Additionally, the anticancer efficacy of MNP@Spiky was further visualized by live and dead assay (Figure 2b). The results showed that tumor cells treated with MNP@Spiky or AMF alone had strong green fluorescence (live cells) and negligible red fluorescence (dead cells), indicating MNP@Spiky had slight toxicity. In contrary, a large number of dead cells were observed in MNP@Spiky+AMF group, demonstrating the tumor‐killing activity of MNP@Spiky with AMF.

To understand the antitumor mechanism of MNP@Spiky, JC‐1 staining assay was carried out. JC‐1 monomers tend to aggregate in healthy mitochondria and form JC‐1 aggregates (red). The occurrence of cell apoptosis can be reflected by the change in the JC‐1‐derived fluorescence from red (JC‐1 aggregates) to green (JC‐1 monomers). As shown in Figure 2c, a decrease in the ratio of red to green was observed in MNP@Spiky+AMF group, confirming the mitochondria‐dependent apoptosis. Moreover, flow cytometry analysis was conducted for quantitative evaluation of cell apoptosis in each group. Consistently, the percentage of apoptotic cells in MNP@Spiky+AMF group was 67.6%, much higher than that in other three groups (Figure 2d,e; Figure S13, Supporting Information).

It is well known that hyperthermia therapy could trigger ICD of tumor cells to induce DC maturation and activate tumor‐specific CTLs. To determine whether MNP@Spiky‐mediated MHT could induce efficient ICD effect, the release or exposure of crucial damage associated molecular patterns (DAMPs), including calreticulin (CRT), high‐mobility group box 1 protein (HMGB1), which are the hallmarks of ICD, were evaluated in the differentially treated groups. As expected, significant translocation of CRT to the cell surface was observed in MNP@Spiky+AMF group (Figure 2f; Figure S14, Supporting Information). The flow cytometric analysis revealed a 5.9‐fold increase in MFI compared with control group. Accordingly, the release of HMGB1 from the nuclear was also detected after treatment with MNP@Spiky+AMF (Figure 2g). The results were further demonstrated by enzyme‐linked immunosorbent assay (ELISA), which suggested that the secretion of HMGB1 increased by 1.8‐fold compared with the counterparts in control group (Figure 2h). These findings confirmed that MNP@Spiky could trigger robust ICD during MHT, which was fundamental for DC maturation and induction of antitumor immune responses.

### 2.3 Immune Stimulation Effect of MNP@Spiky In Vitro

**Figure 3:**
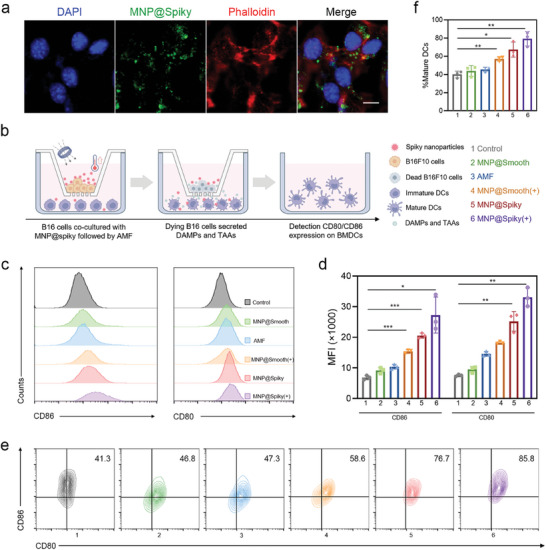
Immune Stimulation Effect of MNP@Spiky In Vitro. a) Immunofluorescence detection of cellular uptake of MNP@Spiky by BMDCs. Scale bar: 10 µm. b) Schematic illustration of the experimental design for co‐culture assay using a Transwell system. Tumor cells were seeded in the upper chamber and BMDCs were placed in the lower chamber. c) Representative histograms of the cell surface markers (CD80 and CD86) on BMDCs after various treatments. d) Quantitative analysis of CD80 and CD86 (MFI) from flow cytometry results in c) (*n* = 3). e) Representative flow cytometric images of DC maturation following different treatments. f) Quantitative analysis of the proportion of mature BMDCs (CD80^+^CD86^+^) from flow cytometry results in e) (*n* = 3). The statistical significance was calculated via Student's *t* test. Data are represented as the mean ± SD. ^*^ *p* < 0.05, ^**^ *p* < 0.01, and ^***^ *p* < 0.001.

DCs play a central role in regulating antitumor immune responses by the uptake of DAMPs and TAAs released by dying tumor cells and the subsequent antigen presentation to activate CD8^+^ T cells. Given the potential immunogenicity of spiky particles, we further investigate their synergistic effect on DC maturation. The cellular uptake of MNP@Spiky was evaluated on bone‐marrow‐derived dendritic cells (BMDCs) isolated from C57BL/6 mice. Strong fluorescence signal was localized with cytoplasm after incubation of FITC‐labeled MNP@Spiky for 12 h, confirming the rapid uptake of MNP@Spiky (**Figure** **3**a).

As illustrated in Figure 3b, a Transwell system was utilized to mimic tumor microenvironment in vivo. B16F10 cells were seeded in the upper chamber for 24 h and treated with PBS, MNP@Smooth, or MNP@Spiky in the presence or absence of AMF for 10 min, then immature BMDCs were added in the bottom chamber and incubated for another 24 h, followed by flow cytometry analysis for DC maturation. The surface markers including CD80 and CD86, which are related to DC maturation, were assessed. MNP@Spiky+AMF group triggered upregulated expression of CD80 (3.98‐fold) and CD86 (4.38‐fold) compared with control group (Figure 3c,d). The percentage of mature DCs increased by 1.54‐fold in BMDCs treated with MNP@Spiky without AMF compared with MNP@Smooth, confirming the immune‐stimulation effect of spiky particles (Figure 3e,f). Moreover, with the assistance of TAAs and DAMPs released by dying B16F10 cells after MHT, MNP@Smooth+AMF group induced 57% of mature DCs, which was 1.3‐fold higher than that of MNP@Smooth group. MNP@Spiky under AMF showed highest proportion of mature DCs, ≈1.8‐, 1.74‐, and 1.39‐fold relative to MNP@Smooth‐, AMF‐, and MNP@Smooth+AMF‐treated groups, respectively, suggesting the synergistic immune‐stimulation action of spiky surface and MNP@Spiky‐mediated MHT. The spiky surface structure of MNP@Spiky promoted the uptake of tumor antigens induced by MHT, thus enhancing the subsequent antitumor immune responses.

### 2.4 Synergistic Antitumor Performance of MNP@Spiky In Vivo

**Figure 4:**
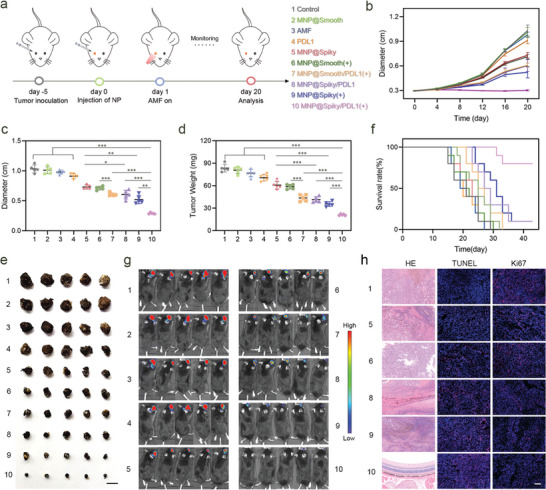
Synergistic Antitumor Performance of MNP@Spiky In Vivo. a) Schematic illustration of the experimental schedule for in vivo antitumor efficacy assessment in orthotopic ocular tumor model. b) Growth curves of orthotopic tumor following different treatments recorded by the diameter of eyeballs. c) Tumor volume represented by the diameter of eyeballs (*n* = 5). d) Tumor weights in various treatment groups (*n* = 5). e) Photographs of dissected eyeballs in each group. Scale bar: 1 cm. f) Survival rates of different groups over time. g) In vivo bioluminescence images of mice after various treatments at day 21. h) Representative images of HE, TUNEL, and Ki‐67 staining in each group. Scale bar: 50 µm. The statistical significance was calculated via Student's *t* test. Data are represented as the mean ± SD. ^*^ *p* < 0.05, ^**^ *p* < 0.01, and ^***^ *p* < 0.001.

Encouraged by the excellent performance of MNP@Spiky in MHT and immunomodulation, the in vivo antitumor activity was evaluated in orthotopic ocular tumor model constructed by subretinal injection of B16F10 cells. Five days later, the mice were injected with PBS, MNP@Smooth, MNP@Spiky, PD‐L1, MNP@Smooth/PD‐L1, or MNP@Spiky/PD‐L1, respectively, followed with or without AMF for 10 min (**Figure** **4**a). To determine the in vivo MHT effect of MNP@Spiky, the real‐time temperature and thermal images of eyeballs were captured by a thermal imaging camera. As shown in Figure S15 (Supporting Information), the temperature of eyeballs in MNP@Spiky group increased and reached 42 °C to realize mild‐temperature MHT and potentiate subsequent immune responses. In contrast, the PBS‐treated group displayed a slight increase with exposure to AMF.

After treatments, the growth of tumor was monitored by the diameter of eyeballs every 4 days for 20 days. Mice treated with MNP@Spiky, MNP@Smooth+AMF, and MNP@Smooth/PD‐L1+AMF slightly delayed tumor growth (Figure 4b). Although MNP@Spiky/PD‐L1 and MNP@Spiky+AMF significantly inhibited tumor growth at the initial stage, the tumors grew rapidly after 12 days. Notably, MNP@Spiky/PD‐L1 under AMF elicited the most robust tumor regression (Figure 4c; Figure S16, Supporting Information). Accordingly, MNP@Spiky/PD‐L1+AMF group showed the lowest tumor weight represented by the eyeball weights (Figure 4d,e). Furthermore, MNP@Spiky/PD‐L1 under AMF significantly prolonged the survival of mice (Figure 4f). Kaplan–Meier survival analysis showed that 80% of mice were still alive in MNP@Spiky/PD‐L1+AMF group after 42 d when mice in other groups are mostly dead. Additionally, the growth of ocular tumor was tracked through the bioluminescence of luciferin (Figure 4g). The results showed MNP@Spiky/PD‐L1+AMF group exhibited the strongest tumor inhibition activity.

The structure of eyeballs was further investigated by hematoxylin‐eosin (HE) staining. As shown in Figure 4h and Figure S17 (Supporting Information), the structure of eyeballs from mice in MNP@Spiky/PD‐L1+AMF group remained basically normal. No discernable tumor tissue was detected, confirming the efficient tumor killing ability of MNP@Spiky/PD‐L1 under AMF. On the contrary, tumor cells massively proliferated and disrupted the eyeball structure in control group. The immunofluorescence staining of Ki‐67 and terminal deoxynucleotidyl transferase‐mediated dUTP‐biotin nick end labeling (TUNEL) staining was performed to investigate the cell proliferation and apoptosis, respectively. The lowest proportion of Ki67‐positive proliferative cell and highest percentage of TUNEL‐positive apoptotic cells were detected in tumor tissue from MNP@Spiky/PD‐L1+AMF group. The results demonstrated that under AMF MNP@Spiky/PD‐L1 significantly inhibited tumor growth and induced tumor cell apoptosis. No apparent toxicity was observed in MNP@Spiky/PD‐L1+AMF group, as evidenced by body weight of mice and HE staining of major organs (heart, liver, spleen, lung, and kidney), indicating the high biosafety and biocompatibility of MNP@Spiky/PD‐L1 (Figure S18, Supporting Information).

### 2.5 MNP@Spiky‐Potentiated Antitumor Immune Responses

**Figure 5:**
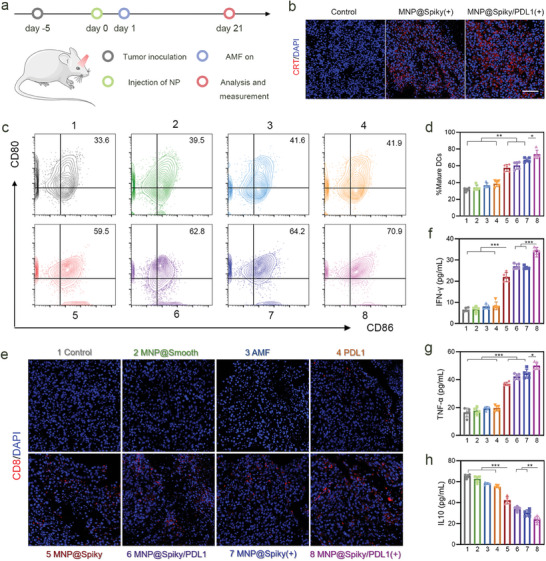
MNP@Spiky‐Potentiated Antitumor Immune Responses. a) Schematic illustration of the experimental schedule for in vivo immune assessment in orthotopic ocular tumor model. b) Representative immunofluorescence staining of CRT in the tumor tissues (*n* = 5). Scale bar: 50 µm. c) Representative flow cytometric images of DC maturation following different treatments. d) Quantitative analysis of the proportion of mature BMDCs in different treatment groups (*n* = 5). e) Representative immunofluorescence staining of CD8^+^ (red) in tumor tissues following various treatments (*n* = 5). Scale bar: 50 µm. f–h) The serum levels of IFN‐γ (f), TNF‐α (g), and IL‐10 (h) detected by ELISA analysis (*n* = 5). The statistical significance was calculated via Student's *t* test. Data are represented as the mean ± SD. ^*^ *p* < 0.05, ^**^ *p* < 0.01, and ^***^ *p* < 0.001.

To elucidate the mechanisms underlying the excellent antitumor performance, the tumor microenvironment was then evaluated by analyzing the immunological processes, including ICD induction, DC maturation, cytotoxic T cell recruitment as well as secretion of cytokines. Mice bearing orthotopic ocular tumor were randomized into eight groups treated with PBS, MNP@Smooth, MNP@Spiky, PD‐L1, or MNP@Spiky/PD‐L1 with or without AMF, respectively (**Figure** **5**a). CRT as the ICD indicator was evaluated by immunofluorescence analysis (Figure 5b; Figure S19, Supporting Information). The strongest expression of CRT was detected in MNP@Spiky/PD‐L1+AMF group. In consistent with in vitro results, MNP@Spiky‐mediated MHT elicited robust ICD and thus initiated subsequent immune responses. Notably, PD‐L1 further augmented the ICD induction effect, possibly due to the increased TAA release after tumor cell killing by cytotoxic T cells.

MNP@Spiky group induced 57.5% mature DCs compared with 33.9% in MNP@Smooth group (Figure 5c,d; Figure S20, Supporting Information). The proportion of mature DCs further increased to 66.9% in MNP@Spiky group under AMF. Encouragingly, with the assistance of ICD induction and PD‐L1 blockade, MNP@Spiky/PD‐L1+AMF group exhibited the synergistic effect on DC maturation, which was 2.17‐, 1.28‐, 1.9, and 1.26‐fold higher than that of MNP@Smooth, MNP@Spiky, PD‐L1, and MNP@Smooth/PD‐L1, respectively. DCs play a crucial role in antigen presentation to T lymphocytes, promoting the recruitment of CTLs (CD8^+^ T cells). Correspondingly, the highest number of CTLs was observed in MNP@Spiky/PD‐L1+AMF group by immunofluorescence staining of tumor sections, as reflected by the red fluorescence signals of the anti‐CD8 antibody, confirming the efficient intratumoral infiltration of CTLs (Figure 5e). Moreover, the serum levels of cytokines including IFN‐γ, TNF‐α, IL10, and IL12 were detected by enzyme‐linked immunosorbent assay (ELISA) kits after different treatments (Figure 5f–h; Figure S21, Supporting Information). IFN‐γ, TNF‐α, and IL12 are key modulators in antitumor immune responses, whereas IL10 is generally immunosuppressive. Consistent with the DC maturation and CTL infiltration, MNP@Spiky/PD‐L1+AMF group showed the highest levels of IFN‐γ, TNF‐α, and IL12, ≈5.3‐, 3.0‐, and 4.2‐fold relative to control, respectively, and lowest level of IL10 (2.7‐fold). Taken together, these results confirmed that MNP@Spiky/PD‐L1+AMF remarkably activated DCs, and thus augmented subsequent antitumor immune responses.

### 2.6 MNP@Spiky‐Induced Inflammasome Activation

**Figure 6:**
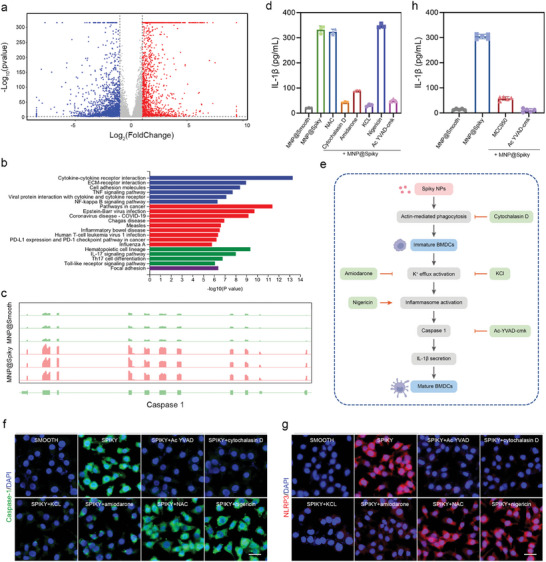
MNP@Spiky‐Induced Inflammasome Activation. a) Volcano plot of identified differentially expressed genes in the MNP@Spiky and MNP@Smooth groups. b) KEGG pathway analysis of the identified differentially expressed genes. c) The expression of caspase‐1 from RNA‐sequence analysis in the MNP@Spiky and MNP@Smooth groups. d) The release of IL‐1β in different groups detected by ELISA. e) Schematic illustration of inflammasome activation mechanism. f,g) Immunofluorescence imaging of caspase‐1 (f) and NLRP3 (g) in BMDCs after different treatments. h) The levels of IL‐1β from mice in various groups detected by ELISA. Data are represented as the mean ± SD.

In view of the prominent ability of MNP@Spiky in DC activation, the potential molecular mechanisms were investigated. The differentially expressed genes of BMDCs between MNP@Spiky and MNP@Smooth‐treated groups were analyzed by RNA sequencing technique. A total of 3917 gene profiles including 1608 upregulated genes and 2309 downregulated genes were identified (**Figure** **6**a; Figure S22, Supporting Information). Gene Ontology (GO) analysis suggested that the differentially expressed genes were enriched in the biological process (BP) of immune‐system process, immune response, and regulation of immune system process, revealing the pivotal role of MNP@Spiky in immune regulation (Figure S23, Supporting Information). Meanwhile, they were enriched in molecular functions (MF) associated with receptor‐binding such as protein binding. Furthermore, Kyoto Encyclopedia of Genes and Genomes (KEGG) pathway analysis showed that MNP@Spiky was mainly involved in cytokine‐cytokine receptor interaction (Figure 6b).

Among the differentially expressed genes, the expression of caspase‐1, which has been reported to be a key factor in the downstream of inflammasome activation pathway, increased by 4.6‐fold (Figure 6c). Inflammasome activation has been demonstrated to be a crucial immune signaling pathway in response to microbes or environmental stress. The NOD‐like receptor protein 3 (NLRP3) inflammasome is one of the largest and most representative inflammasomes, and contains NLRP3, apoptosis‐associated speck‐like protein (ASC), and pro–caspase‐1 protein. The activated caspase‐1 then facilitates the maturation of pro‐interleukin (IL)‐1β into IL‐1β.

To explore how MNP@Spiky triggered the inflammasome activation, the levels of IL‐1β after blocking different signaling pathways were assessed (Figure 6d; Figure S24, Supporting Information). The activity of inflammasome induced by MNP@Spiky in BMDCs was not impeded by N‐acetyl‐l‐cysteine (an inhibitor of reactive oxygen species). On the contrary, cytochalasin D (a phagocytosis inhibitor) completely abolished the activation, indicating the uptake of MNP@Spiky was mediated by phagocytosis. The complete inhibition was also observed after treatment with Ac‐YVAD‐cmk (a caspase‐1 inhibitor), demonstrating the crucial role of caspase‐1 in inflammasome activation. KCl which totally inhibited K^+^ efflux completely abrogated the inflammasome activation. Amiodarone (an inhibitor of K^+^ ion channel) substantially attenuated the release of IL‐1β. These results revealed the central role of K^+^ efflux in inflammasome activation. Moreover, MNP@Spiky induced the inflammasome activation at a similar extent compared with nigericin, a well‐known activator of inflammasome. These findings suggested that the MNP@Spiky was taken up by BMDCs through phagocytosis and induced K^+^‐efflux‐mediated inflammasome activation, followed by cleavage of pro‐caspase‐1 and IL‐1β secretion (Figure 6e). The results were also demonstrated by immunofluorescence staining of caspase‐1 and NLRP3 (Figure 6f,g). In consistence with previous work, the spiky surface could induce membrane curvature and trigger the opening of K^+^ mechanosensitive ion channels which play a role in inflammasome activation.^[^ ^25^ ^]^ The higher mechanical stress might activate K^+^ channel and thus lead to subsequent inflammasome pathway. The activation mechanism was further verified in orthotopic model. As expected, the secretion of IL‐1β was significantly reduced after treatment with MCC950 (a NLRP3 inhibitor) and Ac‐YVAD‐cmk (a caspase‐1 inhibitor) (Figure 6h). Meanwhile, MCC950‐ and Ac‐YVAD‐cmk‐treated groups exhibited similar tumor growth pattern compared with MNP@Smooth‐treated group, which further confirmed that the deletion of either NLRP3 or caspase‐1 abolished the tumor inhibition effect induced by MNP@Spiky‐mediated immune activation (Figure S25, Supporting Information).

## 3 Conclusion

In summary, a novel therapeutic strategy based on MNP@Spiky was developed for inducing MHT and immunotherapy to circumvent current obstacles in UM treatment, where the immunosuppressive microenvironment with lower immunorecognition and immune cell infiltration hindered the efficacy of cancer immunotherapy. Owing to the MHT capacity of Fe_3_O_4_ core, MNP@Spiky not only attacked tumor cells by thermal ablation, but also induced ICD to initiate antitumor immune responses. Decorated with a spiky surface, MNP@Spiky posed more mechanical stress on the membrane of DCs, which resulted in the K^+^ efflux and inflammasome activation in a caspase‐1‐dependent manner and significantly promoted the maturation and antigen uptake of DCs. Notably, the obvious immune activation induced by the high immunogenicity of spiky surface nanostructure provided a novel avenue to reverse the immunosuppressive tumor microenvironment and augment the efficacy of immunotherapy. Furthermore, upon addition with PD‐L1, the combined system rescued CTLs from dysfunction and enormously bolstered antitumor immune responses. Our findings demonstrated that this synergistic therapeutic strategy triggered robust antitumor immunity and achieved conspicuous tumor inhibition in vitro and in vivo. Overall, this study not only provided an appealing option for UM treatment by synergistic MHT and immunotherapy, but also offered new insights into the rational design of nanoplatform for efficient cancer immunotherapy.

## Conflict of Interest

The authors declare no conflict of interest.

## Supporting information



Supporting Information

## Data Availability

The data that support the findings of this study are available from the corresponding author upon reasonable request.
