# Supplementary material for: Unlocking the Promise of Antitumor Hyperthermia‐Immunotherapy with Spiky Surface Topology
Source: Adv Sci (Weinh). 2025 Feb 18;12(14):2415868. doi: 10.1002/advs.202415868 (PMC11984835; doi:10.1002/advs.202415868)
Supplement: Supplementary file 1 — Supporting Information [file ADVS-12-2415868-s001.docx]

**Supporting Information**

**Unlocking the Promise of Antitumor Hyperthermia-immunotherapy with Spiky Surface Topology**

Muyue Yang^1,2,3^, Yan Yu^4^, Tongxin Ge^1,2,3^, Qiuyi Zhu^1,2,3^, Ai Zhuang^1,2,3^, Wenxing Wang^4^, Xianqun Fan^1,2,3^

**Affiliation**

^1^ Department of Ophthalmology, Shanghai Ninth People’s Hospital, Shanghai JiaoTong University School of Medicine, Shanghai 200011, China
^2^ State Key Laboratory of Eye Health, Shanghai Jiao Tong University
^3^ Shanghai Key Laboratory of Orbital Diseases and Ocular Oncology, Shanghai 200011, China

^4^ Department of Chemistry, State Key Laboratory of Molecular Engineering of Polymers, Laboratory of Advanced Materials, Fudan University, Shanghai 200433, China

**Correspondence to:**

Ai Zhuang^a^, Wenxing Wang^b^, Xianqun Fan^c^

^a^ E-mail: aizh9h@163.com

^b^ E-mail: wangwenxing@fudan.edu.cn

^c^ E-mail: [fanxq@sjtu.edu.cn](mailto:fanxq@sjtu.edu.cn)

**1. Method**

Synthesis of MNP (Fe_3_O_4_ nanoparticles)

For the synthesis of MNP, FeCl_3_∙6H_2_O (1.62 g), trisodium citrate (0.65 g), and NaAc (3.0 g) were dissolved in ethylene glycol (50 mL) under vigorous stirring. Then, the obtained solution was transferred into a Teflon-lined autoclave (100 mL) and heated at 180 °C for 12 h. Finally, the products were collected and washed with deionized water and ethanol for several times by centrifugation or magnetic separation, and then dried under vacuum at 60 °C for further use.

Synthesis of the MNP@Smooth

For the synthesis of MNP@Smooth, a modified Stöber method was applied. Generally, Fe_3_O_4_ (40 mg) was dispersed in a solution containing ethanol (80 mL), deionized water (10 mL), and aqueous ammonia (1.8 mL), with stirring for 1 h. Then, TEOS (2 mL) was added to the above system with stirring for another 8 h. The products were collected by centrifugation and washed with water and ethanol several times.

Synthesis of the MNP@Spiky

MNP@Spiky were synthesized by the single-micelle epitaxial growth method in a biphase system as previously reported. Typically, MNP (60 mg) was dispersed in water (50 mL) by sonication. Afterward, CTAB (1.5 g) and 0.8 mL of NaOH (0.1 m) were added to the solution under gentle stirring (≈300 rpm) at 60 °C for 2 h. Then, the mixture (20 mL) of TEOS (4 mL) in cyclohexane (16 mL) was added to the above solution and kept at 60 °C with slow stirring (≈300 rpm) for 48 h. The obtained MNP@Spiky samples were collected by centrifugation and washed with water and ethanol for several times.

Synthesis of Amino Group-Modified MNP@Smooth and MNP@Spiky

The amino groups were grafted on the core-shell nanoparticles by post-synthetic modification method. First, the MNP@Smooth or MNP@Spiky sample (100 mg) was dispersed in ethanol (40 mL) by ultrasonic. Then, 3-aminopropyltriethoxysilane (200 µL) was added, and the solution was refluxed for 24 h under the nitrogen atmosphere with continuous stirring. The products were collected by centrifuging and washed with ethanol, and water, and dried in vacuum at 40 °C for 24 h.

Synthesis of FITC-Modified MNP@Smooth and MNP@Spiky

For the synthesis of FITC-labeled smaples, amino group-modified samples (20 mg) were dispersed in ethanol (10 mL), and 1 mL of FITC ethanol solution (0.5 mg/mL) was added. The reaction mixture was stirred for 12 h in the dark. The products were then separated by centrifugation. After washed with ethanol and deionized water for several times, the samples were redispersed in aqueous solution.

Characterization of MNP@Smooth and MNP@Spiky

Transmission electron microscopy (TEM) images were acquired on a JEM-1400 microscope operating at 120 kV. The morphologies of the samples were further analyzed using a field-emission scanning electron microscope (FE-SEM, S4800, Hitachi). High-resolution TEM (HRTEM) images, high-angle annular dark-field (HAADF) image and energy-dispersive X-ray spectroscopy (EDS) mapping were obtained on JEM-2100F microscope (JEOL, Beijing, China) with an accelerating voltage of 200 kV equipped with a post-column Gatan imaging filter. The phase of MNP@Spiky was characterized by a Bruker D8 powder X-ray diffractometer (Germany) with Ni-filtered Cu Kα radiation (40 kV, 40 mA). The content of Fe in MNP@Spiky and MNP@Smooth were determined by an inductively coupled plasma emission spectroscopy instrument (ICP, Thermo Scientific, iCAP 7000 Series). The hydrodynamic size of the samples was determined by a Malvern Zeta sizer Nano ZS instrument. The magnetization curves of MNP@Smooth and MNP@Spiky were measured by vibrating sample magnetometer at room temperature. The magnetic hyperthermia property of the samples (Fe concentration: 1 mg mL^-1^) with the frequency of 410 kHz were recorded by thermal imaging system.

Cell culture conditions and animals

B16F10 cells (melanoma cell line) were obtained from the Cell Bank of the Chinese Academy of Sciences, Shanghai, China, and cultured in Roswell Park Memorial Institute 1640 medium (RPMI 1640; Invitrogen, Carlsbad, CA, USA) with 10% fetal bovine serum (FBS; Gibco, USA), penicillin (100 units/mL), and streptomycin (100 μg/mL). BMDCs were isolated from bone marrow cells from C57BL/6 male mice (8 weeks old) according to a well-established protocol.^[1]^ Briefly, femurs and tibiae of mice were isolated and the single-cell suspension was obtained by the flush of bone marrow. The obtained BMDCs were cultured in RPMI 1640 containing 10ng/mL GM-CSF for 7 days.

C57BL/6 mice were purchased from Shanghai SLAC Laboratory Animal Co., Ltd. and kept in the China State Institute of Pharmaceutical Industry and Shanghai Model Organisms Center, Inc. All animal experiments were approved by the Animal Research Committee of Shanghai Ninth People’s Hospital, Shanghai Jiaotong University School of Medicine, and the Animal Research Committee of Shanghai Model Organisms Center, Inc.

Cellular uptake

To investigate the cellular uptake of nanoparticles, B16F10 cells and BMDCs were seeded in 24-well plates (1 × 10^5^ cells per well) and treated with 100 μg/mL fluorescein isothiocyanate (FITC)-labeled MNP@Spiky for 12 h. Then the cells were fixed, collected, and detected by confocal laser scanning microscope (CLSM) and Bio- transmission electron microscope (TEM).

Cell viability assay

B16F10 cells were seeded into 96-well plates at a density of 1 × 10^5^ cells per well with RMPI 1640 medium for 12 h. Then, MNP@Spiky at different concentration was added and incubated for 2 h. Then the cells were implemented with or without AMF (1.7 mT) for 10 min. The cell viability was detected by a cell counting kit-8 (CCK-8) assay (Dojindo Molecular Technologies, Inc., Japan). 1 × 10^4^ B16F10 cells per well were seeded in 96-well plates. The CCK8 solution was diluted by cell medium at a ratio of 1:10 and added to the medium. The absorbance was measured by a microplate reader (ELX800, BioTec, Winooski, VT, United States) at a wavelength of 450 nm after incubation for 4 hours at 37 °C.

For live/dead staining, B16F10 cells were seeded (1 × 10^5^ cells per well) in 24-well plate overnight and incubated with or without MNP@Spiky (5 mg/mL). Then B16F10 cells were treated with or without AMF (1.7 mT, 10 min). PBS containing calcein-AM and propidium iodide were used to replace the cell media. After staining for 30 min, the images were taken by fluorescence microscope (Olympus BX51, Japan).

For apoptosis analysis, B16F10 cells were seeded into a 6-well culture plate (5 × 10^5^ cells per well) overnight. After different treatments cells were collected and stained with Annexin V-FITC/PI kit (BD Biosciences, USA). The cell apoptosis was analyzed by flow cytometry (BD Biosciences, USA) using FlowJo software for acquisition and analysis.

For mitochondrial membrane potential assay, B16F10 cells were seeded into a 24-well culture plate (1 × 10^5^ cells per well). The mitochondrial membrane potential was evaluated by MitoProbe JC-1 assay kit (Thermo Fisher Scientific–US) and then imaged by laser scanning confocal microscopy. JC-1, a mitochondrial dye, can change color from red to green as the membrane potential decreasing. JC-1 tends to aggregate in healthy mitochondria and emits red fluorescence. When the mitochondrial integrity is destroyed, JC-1 forms monomer and emits green fluorescence. JC-1 aggregate: λex: 561 nm, λem: 560–610 nm; JC-1 monomer: λex: 488 nm, λem: 500–550 nm.

*In vitro* immunologic cell death (ICD) detection

To evaluate ICD induction, the surface expression of CRT and the release of HMGB1 were detected *in vitro*. For immunofluorescence staining of CRT expression, B16F10 cells after various treatments were stained with anti-mouse CRT antibody at 4 ℃ overnight, and then stained with Alexa Fluor 488 donkey anti-rabbit IgG secondary antibody at room temperature for 1 h. Then the cells were stained with DAPI and examined by CLSM. For immunofluorescence detection of HMGB1 release, B16F10 cells in different groups were stained with anti-mouse HMGB1 antibody at 4 ℃ overnight, and then stained with Alexa Fluor 568 donkey anti-rabbit IgG secondary antibody at room temperature for 1 h. Then the cells were stained with DAPI and examined by CLSM. For flow cytometric analysis of CRT expression on cell membrane, cells after various treatments were collected and stained with anti-CRT antibody at 4 ℃ for 30 min, followed by an Alexa Fluor 568 secondary antibody at 4 ℃ for 30 min, and then the cells were analyzed by flow cytometry (BD Biosciences, USA). For detection HMGB1 release, the supernatants from cells in each group were collected and tested by HMGB1 ELISA kit.

*In vitro* evaluation of BMDC maturation

A Transwell system was applied to mimic tumor microenvironment *in vivo*. B16F10 cells were seeded in the upper compartment for 24 h and treated with PBS, MNP with a smooth surface (MNP@Smooth, 5 mg/mL), or MNP@Spiky (5 mg/mL) with or without of AMF for 10 min, then immature BMDCs were seeded in the bottom chamber and incubated for another 24 h. Then BMDCs were collected and stained with anti-CD11c, anti-CD80, and anti-CD86 at 4 ℃ for 30 min, followed by flow cytometry analysis (BD Biosciences, USA) for DC maturation.

*In vivo* anti-tumor evaluation in orthotopic ocular tumor model

The orthotopic ocular tumor model was established by injection of five microliters of medium containing 2 × 10^5^ B16F10 cells into the sub-retinal cavities of C57BL/6 mice (6-8 weeks, female). B16F10 tumor-bearing C57BL/6 mice were then randomly divided into ten groups, including control, MNP@Smooth, AMF, PDL1, MNP@Spiky, MNP@Smooth(+) (MNP@Smooth with AMF), MNP@Smooth/PDL1(+) (MNP@Smooth/PDL1 with AMF), MNP@Spiky/PDL1, MNP@Spiky(+) (MNP@Spiky with AMF), MNP@Spiky/PDL1 (+) (MNP@Spiky/PDL1 with AMF). Five days after tumor implantation, the mice were injected with 5 μL of sterile PBS, MNP@Smooth (5 mg/mL), MNP@Spiky (5 mg/mL), PD-L1 (100 µg/mL), MNP@Smooth/PD-L1 (MNP@Smooth, 5 mg/mL; PD-L1, 100 µg/mL), or MNP@Spiky/PD-L1 (MNP@ Spiky, 5 mg/mL; PD-L1, 100 µg/mL), respectively, followed with or without AMF for 10 min after 24 h of administration. The real-time temperature and thermal images of eyeballs were captured by a thermal imaging camera. Then the diameter of eyeballs was recorded every four days. The IVIS Lumina system was applied to monitor the growth of orthotopic tumors. The body weight of mice was recorded. Mice were sacrificed on day 20 after treatment, and eyeballs were collected for hematoxylin-eosin (HE) and Ki-67 staining, and terminal-deoxynucleoitidyl transferase mediated nick end labeling (TUNEL) staining. The other major organs were harvested for HE staining.

Tumor immune microenvironment analysis

B16F10 tumor-bearing C57BL/6 mice were randomly divided into eight groups, including control, MNP@Smooth, AMF, PDL1, MNP@Spiky, MNP@Spiky/PDL1, MNP@Spiky (+) (MNP@Spiky with AMF), MNP@Spiky/PDL1 (+) (MNP@Spiky/PDL1 with AMF). Five days after tumor implantation, the mice were treated with PBS, MNP@Smooth (5 mg/mL), MNP@Spiky (5 mg/mL), PD-L1 (100 µg/mL), or MNP@Spiky/PD-L1 (MNP@ Spiky, 5 mg/mL; PD-L1, 100 µg/mL) with or without AMF, respectively. On day 21 post injection, the eyeballs and blood of mice were collected for further study. For ICD evaluation, eyeballs were stained with anti-CRT antibody, followed by staining of secondary antibody (CY3-goat anti-rabbit IgG) and detection of CLSM. For DC maturation evaluation, eyeballs were digested with collagenase type IV, deoxyribonuclease I and hyaluronidase to obtain single-cell suspensions. Then the single-cell suspension was collected and stained with antibodies against CD11c, CD80, and CD86, followed by flow cytometry analysis (BD Biosciences, USA) for DC maturation. For CTL evaluation, eyeballs were stained with anti-CD8 antibody, followed by staining of secondary antibody (CY3-goat anti-rabbit IgG) and detection of CLSM. For detection of cytokine release, the serum level of INF-γ (R&D, Catalog: MIF00), TNF-α (R&D, Catalog: MTA00B), IL-10 (R&D, Catalog: M1000B), and IL-12 (R&D, Catalog: M1270) were analyzed with enzyme-linked immunosorbent assay (ELISA) kits according to the manufacturer’s instructions. The OD value of each sample was detected. The serum concentration was calculated according to the OD values based on standard curve.

RNA sequencing

Total RNA was extracted using the Trizol Reagent (Invitrogen Life Technologies). The purity and quality of RNA were evaluated using a NanoDrop spectrophotometer (Thermo Scientific). Then the sequencing libraries were generated by the TruSeq RNA Sample Preparation Kit (Illumina, San Diego, CA, USA) according to the manufacturer’s instructions. In brief, mRNA was purified from total RNA by magnetic beads linked with poly-T oligonucleotides. Divalent cations were used for fragmentation in the Illumina proprietary fragmentation buffer at elevated temperature. The first strand cDNA synthesis was performed during random oligonucleotides and SuperScript II. The second strand cDNA was then synthesized by DNA Polymerase I and RNase H. The remaining overhangs were converted into blunt ends by the activity of exonuclease/polymerase. After the 3′ end adenylation, Illumina PE adapter oligonucleotides were connected to prepare for hybridization. To select preferred cDNA fragments with a length of 200 bp, the library fragments were purified by the AMPure XP system (Beckman Coulter,Beverly, CA, USA). In 15 cycles of PCR reaction, Illumina PCR Primer Cocktail was applied to selectively enrich the DNA fragments with ligated adaptor molecules on both ends. Then the products were purified and quantified by the high sensitivity DNA assay on Agilent Bioanalyzer 2100 system. The sequencing library was then sequenced on an Illumina Novaseq platform. An absolute fold change of > 2 or < 0.5 was considered as differentially expressed genes.

Inflammasome activation

BMDCs were cultured with MNP@Spiky or MNP@Smooth for 12 h. To explore the mechanisms undrlying the inflammasome activation, the levels of IL-1β after blocking different signaling pathways were assessed. The inhibitor or activator, including Ac-YVAD-cmk (50 μM), nigericin (3 μM), cytochalasin D (2 μM), KCl (130 mM), amiodarone (10 μM), N-acetyl-L-cysteine (NAC, 5 mM), was added to BMDCs 30 min prior to MNP@Spiky treatment. Then, the concentration of IL-1β after different treatments was detected by ELISA kit (eBioscience). For immunofluorescence staining of caspase-1, cells after various treatments were stained with anti-caspase-1 antibody at 4 ℃ overnight, and then stained with Alexa Fluor 488 donkey anti-rabbit IgG secondary antibody at room temperature for 1 h. Then the cells were stained with DAPI and examined by CLSM. For immunofluorescence detection of NLRP3, cells after different treatments were stained with anti-NLRP3 antibody at 4 ℃ overnight, and then stained with Alexa Fluor 568 donkey anti-rabbit IgG secondary antibody at room temperature for 1 h. Then the cells were stained with DAPI and examined by CLSM.

For *in vivo* evaluation, B16F10 tumor-bearing C57BL/6 mice were randomly divided into four groups, including MNP@Smooth, MNP@Spiky, MNP@Spiky+MCC950 (NLRP3 inhibitor, 10 μM), MNP@Spiky+Ac-YVAD-cmk (caspase-1 inhibitor, 50 μM). The diameter of eyeballs was recorded to evaluate the tumor volume. The IVIS Lumina system was applied to monitor the growth of orthotopic tumors.

Statistical Analysis

All data are expressed as the mean ± standard deviation. Statistical evaluation was analyzed by unpaired two-tailed Students’ *t*-test using GraphPad Prism software. Results with P value ≤ 0.05 were regarded as statistically significant (**p* < 0.05, ***p* < 0.01, and ****p* < 0.001).

**2. Supplementary figures**

**Figure S1**. Hydrodynamic size distribution of MNP@Spiky.


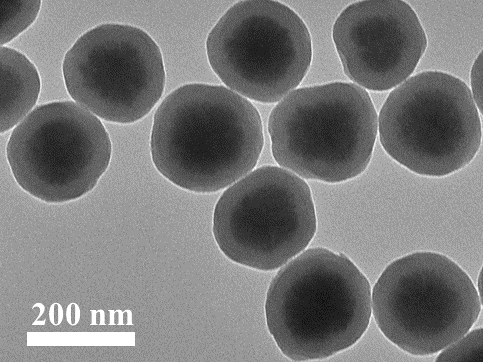


**Figure S2**. TEM image of MNP@Smooth.


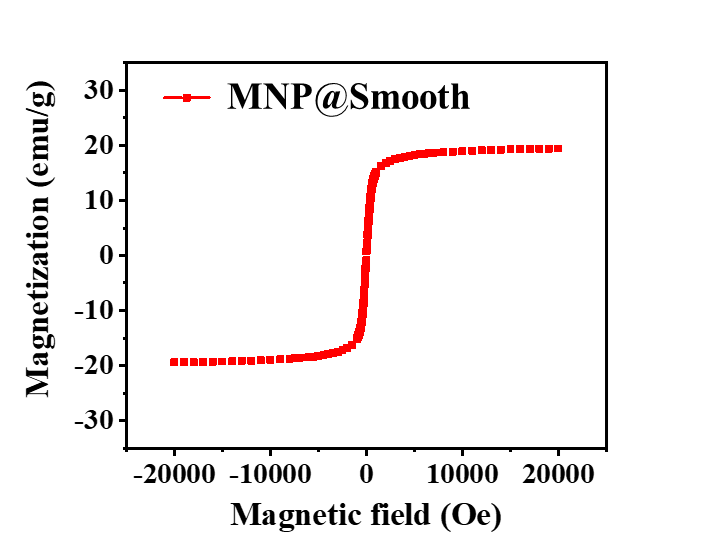


**Figure S3**. The magnetization curve of MNP@Smooth.


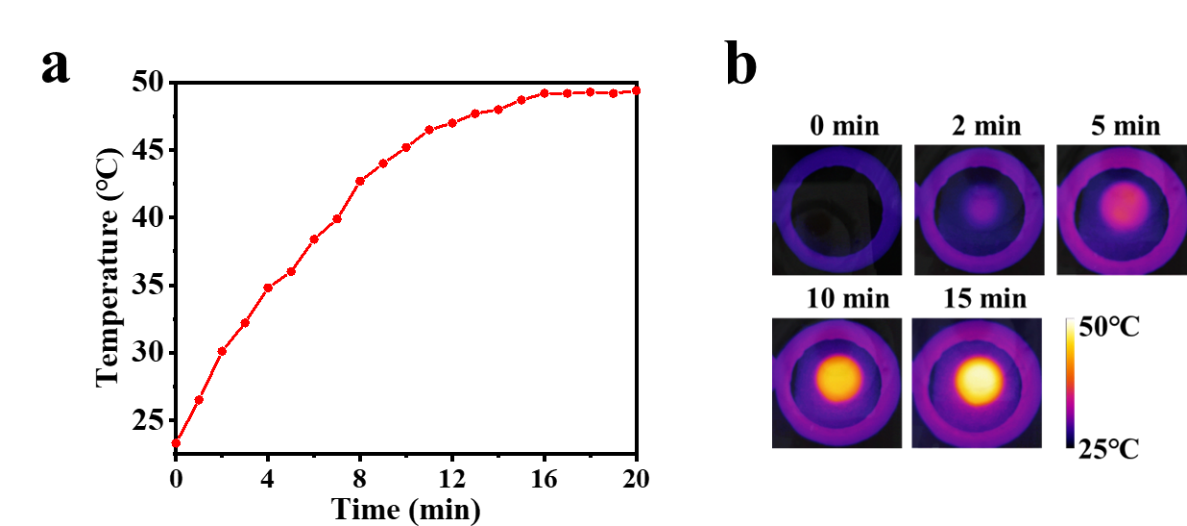


**Figure S4**. Heating profiles of MNP@Smooth. (a) Temperature–time curve and (b) real time *in vivo* IR thermal images of MNP@Smooth.


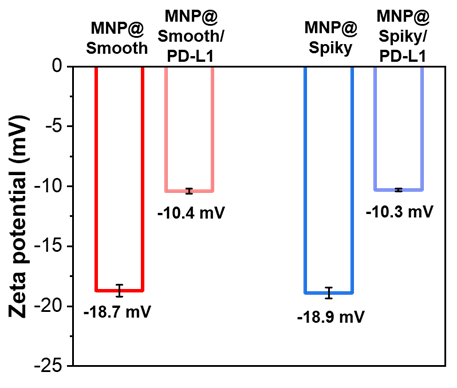


**Figure S5.** Zeta potential analysis of MNP@Smooth, MNP@Smooth/PD-L1, MNP@Spiky and MNP@Spiky/PD-L1 in PBS solution.


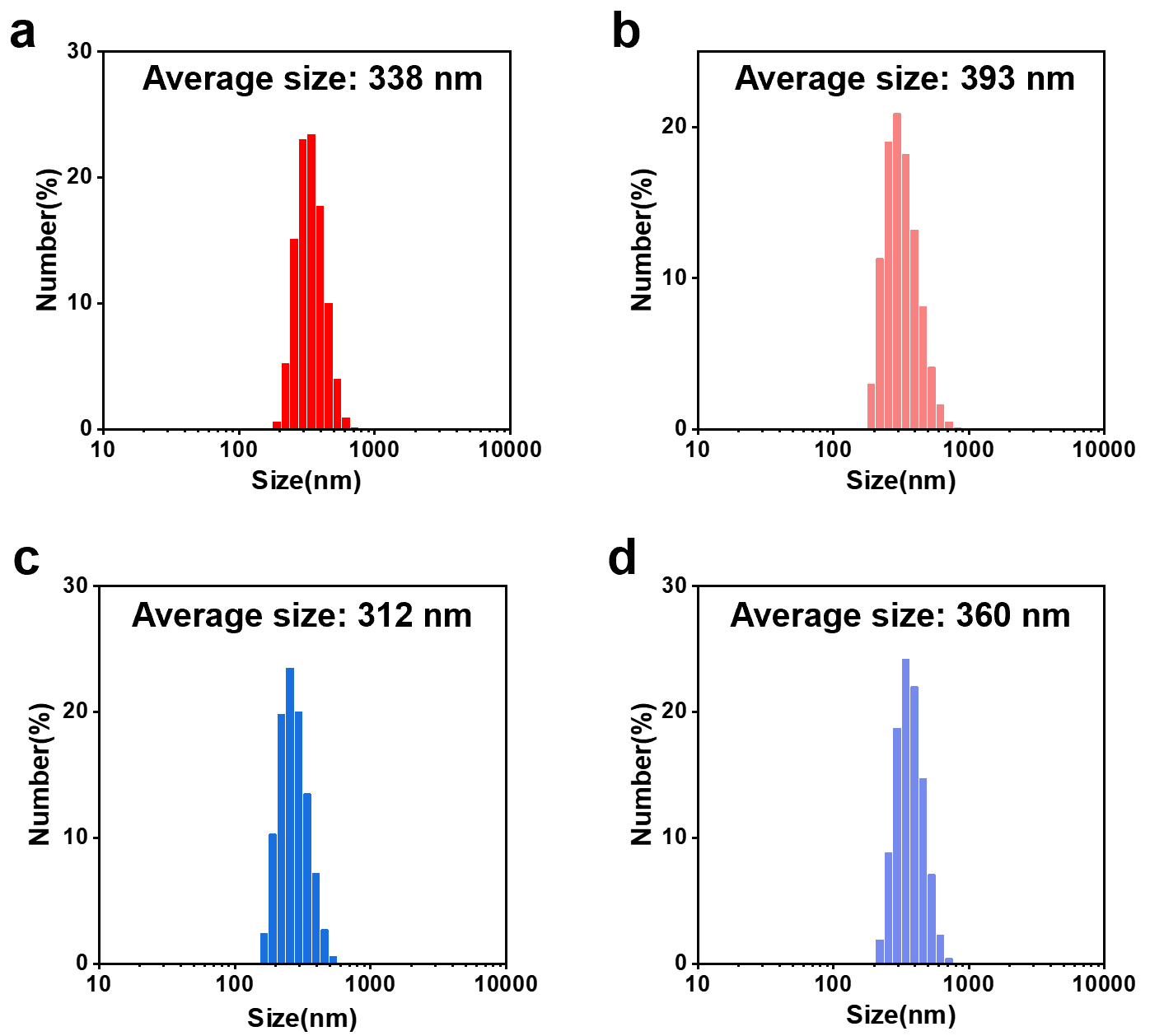


**Figure S6**. (a) Hydrodynamic size distribution of MNP@Smooth in PBS solution. (b) Hydrodynamic size distribution of MNP@Smooth/PD-L1 in PBS solution. (c) Hydrodynamic size distribution of MNP@Spiky in PBS solution. (d) Hydrodynamic size distribution of MNP@Spiky/PD-L1 in PBS solution.


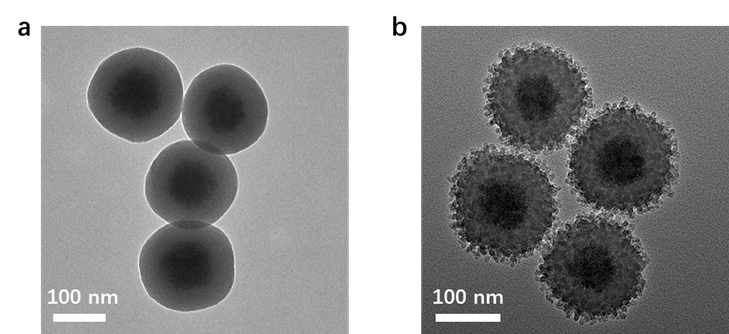


**Figure S7.** TEM images of (a) MNP@Smooth and (b) MNP@Spiky after associating with PD-L1. For TEM sample preparation, these samples were cleaned and dispersed in ethanol before dropping on the grids.


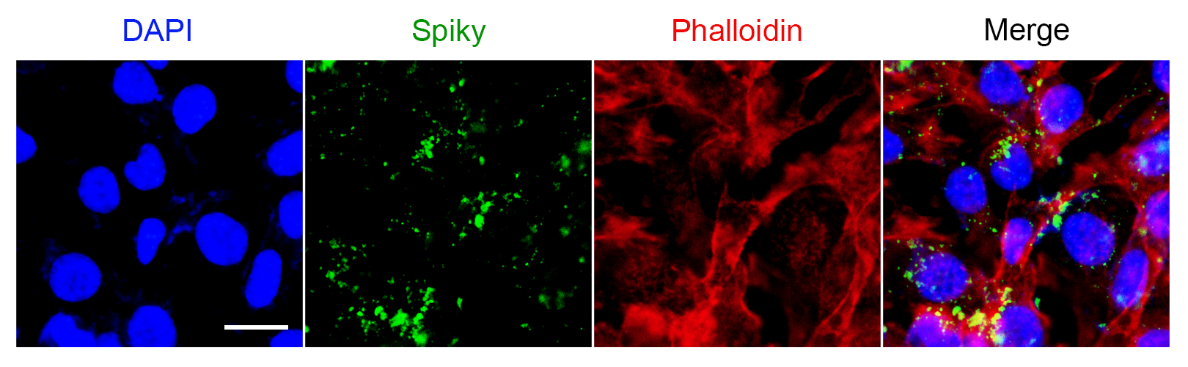


**Figure S8**. Immunofluorescence detection of cellular uptake of MNP@Spiky by tumor cells. Scale bar: 10 µm.


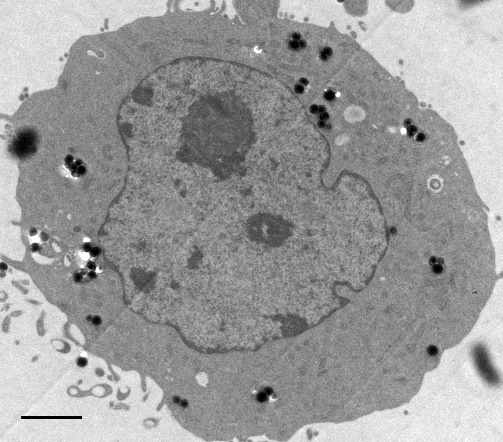


**Figure S9**. Bio-TEM images of cellular uptake of MNP@Spiky by tumor cells. Scale bar: 2 µm.


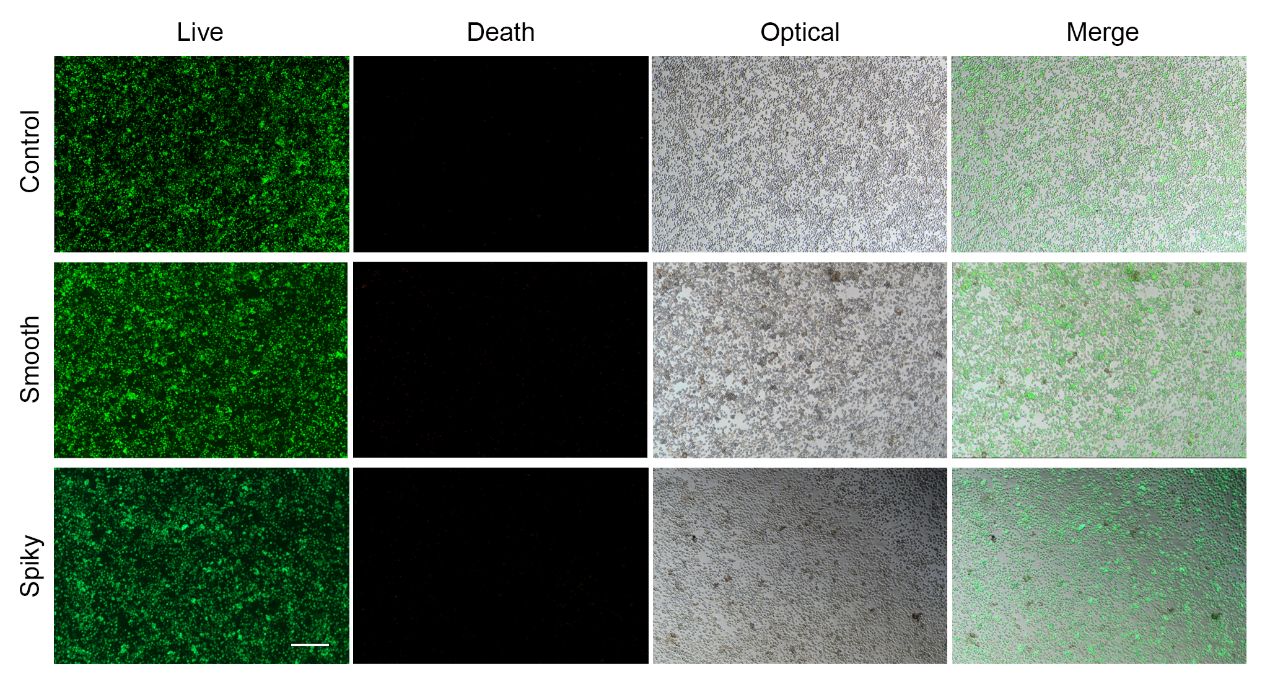


**Figure S10**. Fluorescence and optical images of NIH/3T3 cells after incubation with MNP@Smooth and MNP@Spiky. Green, live cells; Red, dead cells. Scale bar: 200 μm.


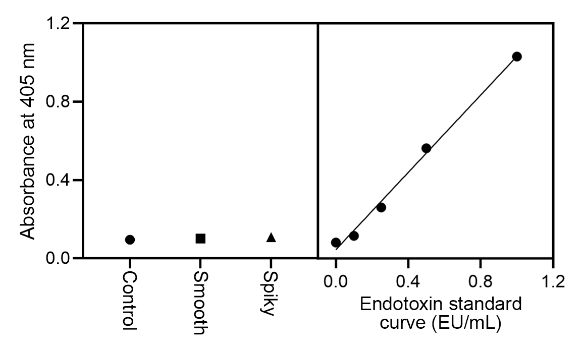


**Figure S11**. The endotoxin levels of MNP@Smooth and MNP@Spiky were not significantly higher compared to the endotoxin-free control.


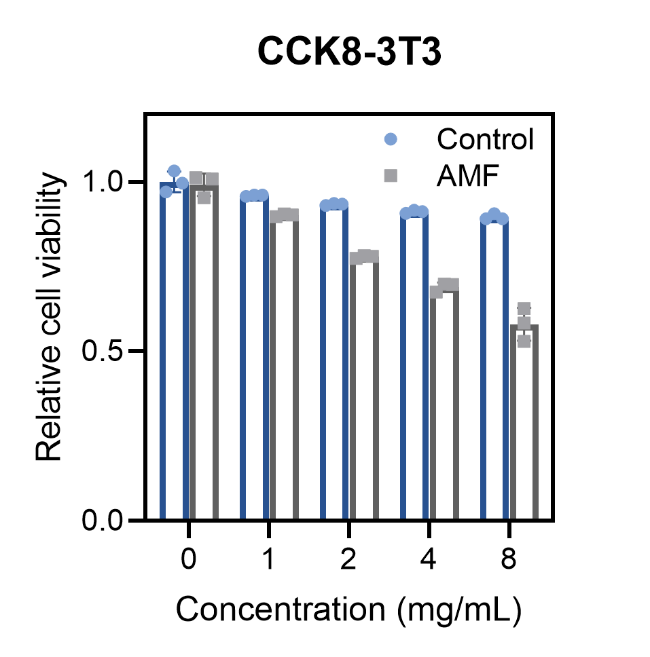


**Figure S12**. Relative cell viability of NIH/3T3 cells after treatment with different concentration of MNP@Spiky, followed with or without AMF.


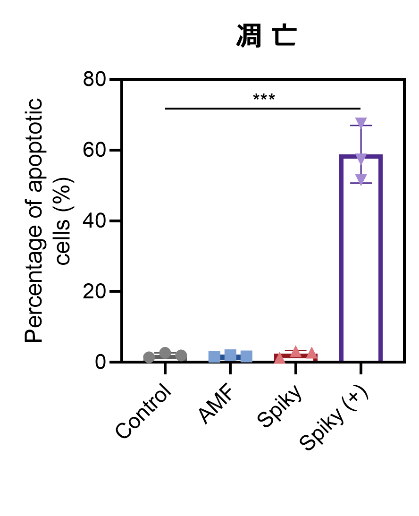


**Figure S13**. Quantitative analysis of cell apoptosis from flow cytometry results in Figure 2d (n = 3). The statistical significance was calculated via Student’s *t* test. Data are represented as the mean ± SD. ****p* < 0.001.


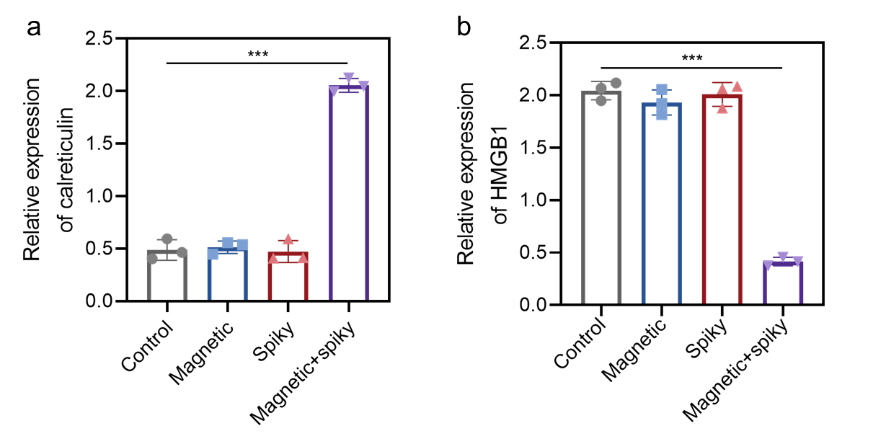


**Figure S14**. Quantitative analysis of the expression of CRT and HMGB1 from immunofluorescence staining results in Figure 2f (n = 3). The statistical significance was calculated via Student’s *t* test. Data are represented as the mean ± SD. ****p* < 0.001.


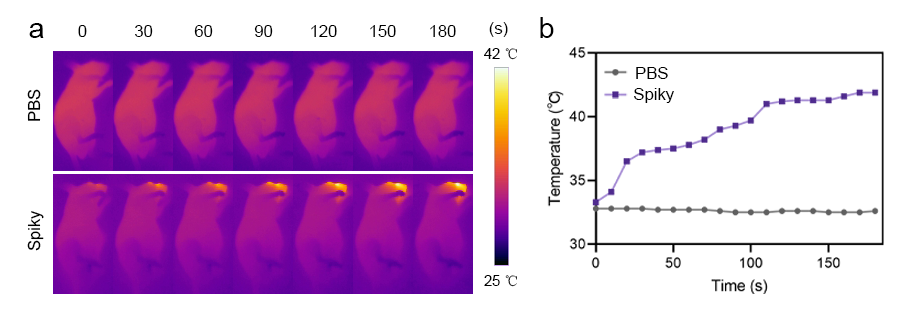


**Figure S15**. a) Infrared thermal images of the mice injected with PBS or MNP@Spiky in response to AMF. b) Temperature of the tumors following different treatments.


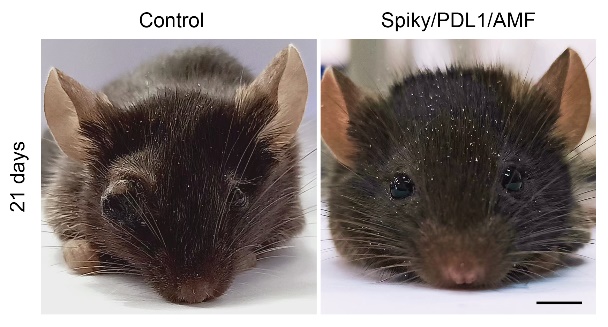


**Figure S16**. Representative images of mice in control and MNP@Spiky/PDL1(+) group.


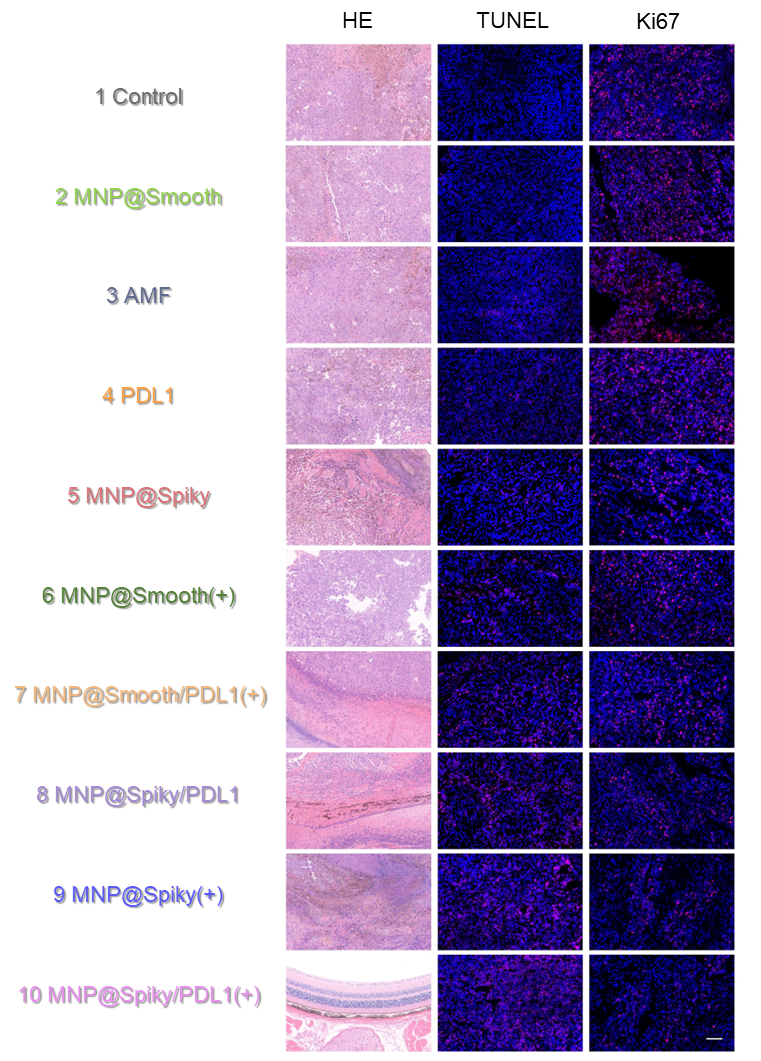


**Figure S17**. Representative images of HE, TUNEL, and Ki-67 staining in each group. Scale bar: 50 µm.


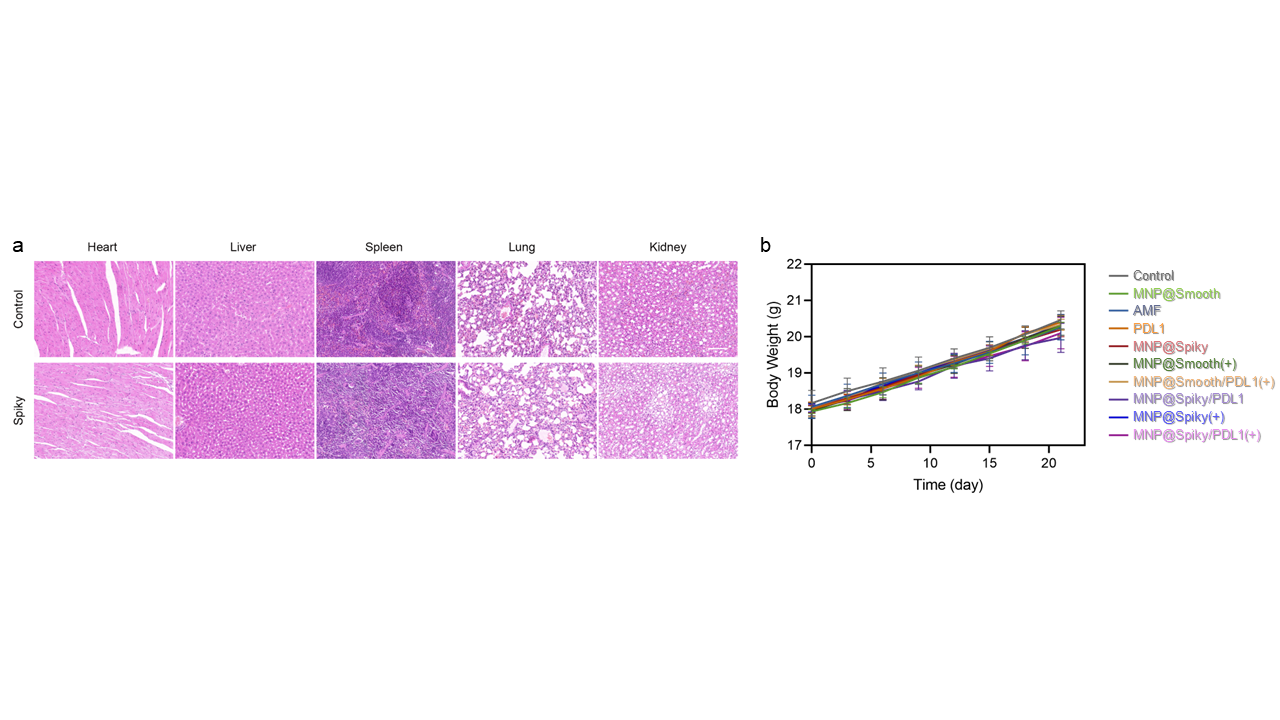


**Figure S18**. Biosafety evaluation of MNP@Spiky/PD-L1. a) No apparent toxicity was observed in HE images of heart, liver, spleen, lung, and kidney. Scale bar: 100 µm. b) The body weight of mice in each group. No significant difference was observed between different groups.


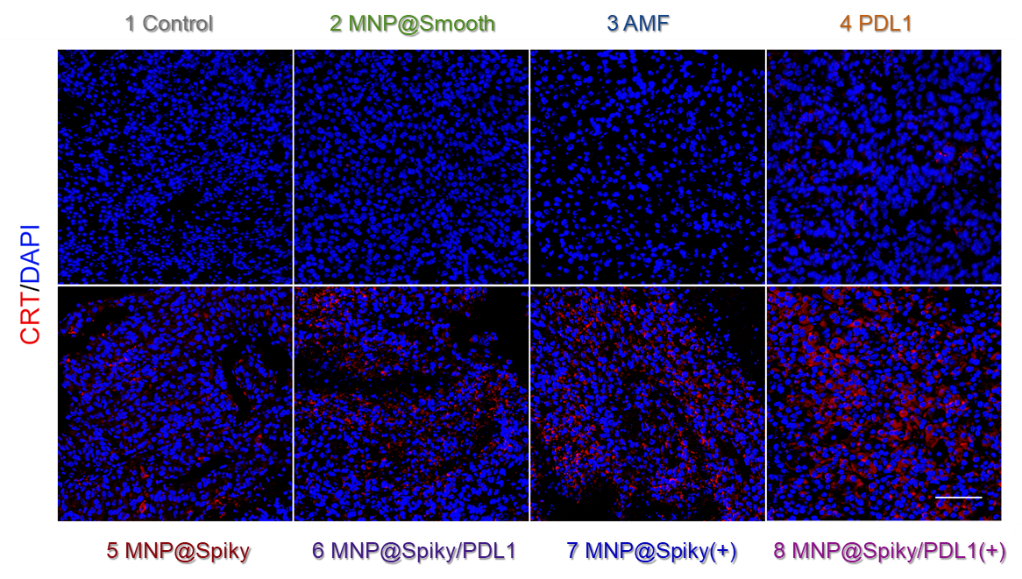


**Figure S19**. Immunofluorescence staining of CRT in the tumor tissues (n = 5). Scale bar: 50 µm.


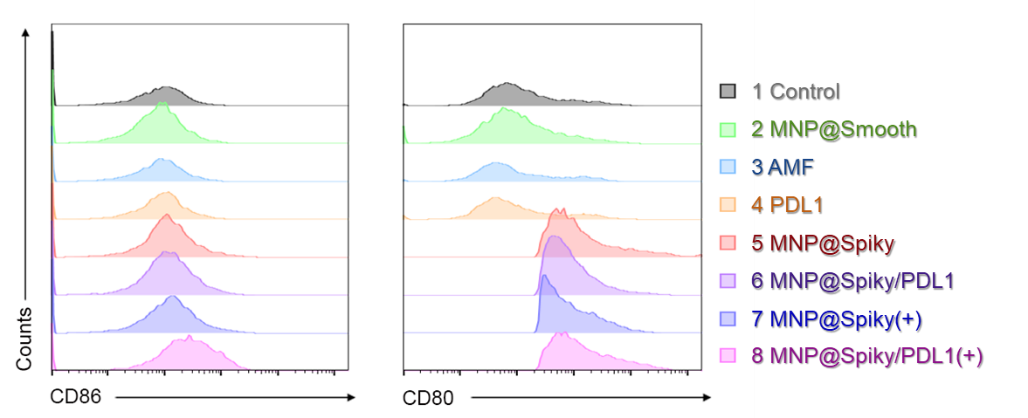


**Figure S20**. Representative histograms of the cell surface markers (CD80 and CD86) on DCs in different groups.


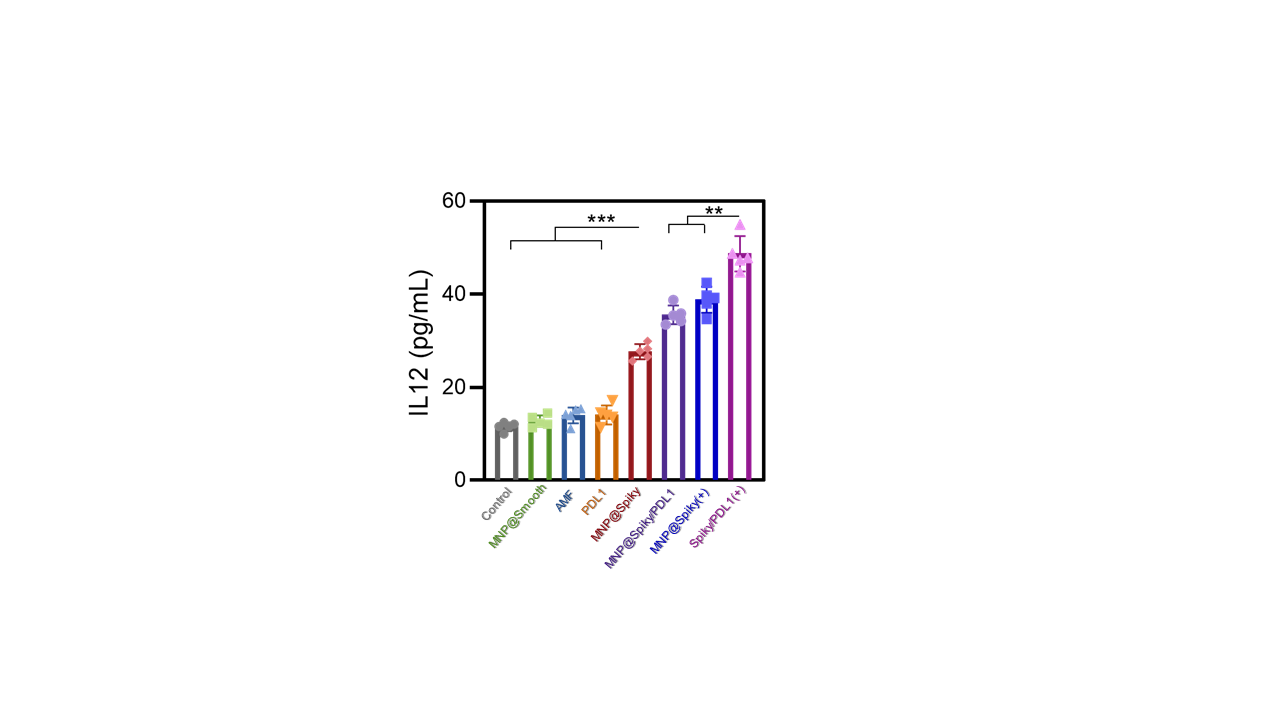


**Figure S21**. The serum levels of IL-12 detected by ELISA analysis (n = 5). The statistical significance was calculated via Student’s *t* test. Data are represented as the mean ± SD. ***p* < 0.01, ****p* < 0.001.


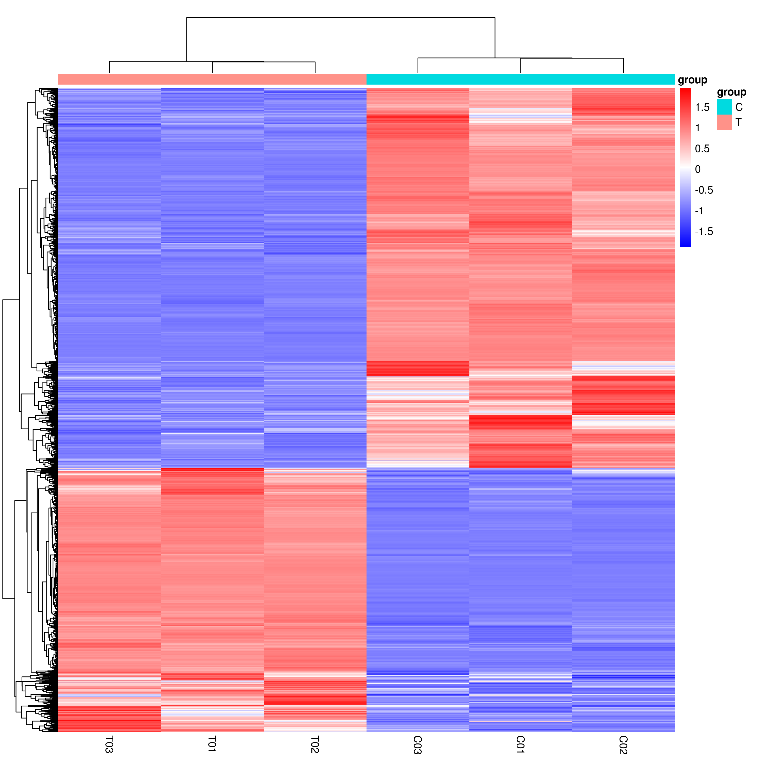


**Figure S22**. Heatmap of identified differentially expressed genes in the MNP@Spiky (T) and MNP@Smooth (C) groups (n = 3).


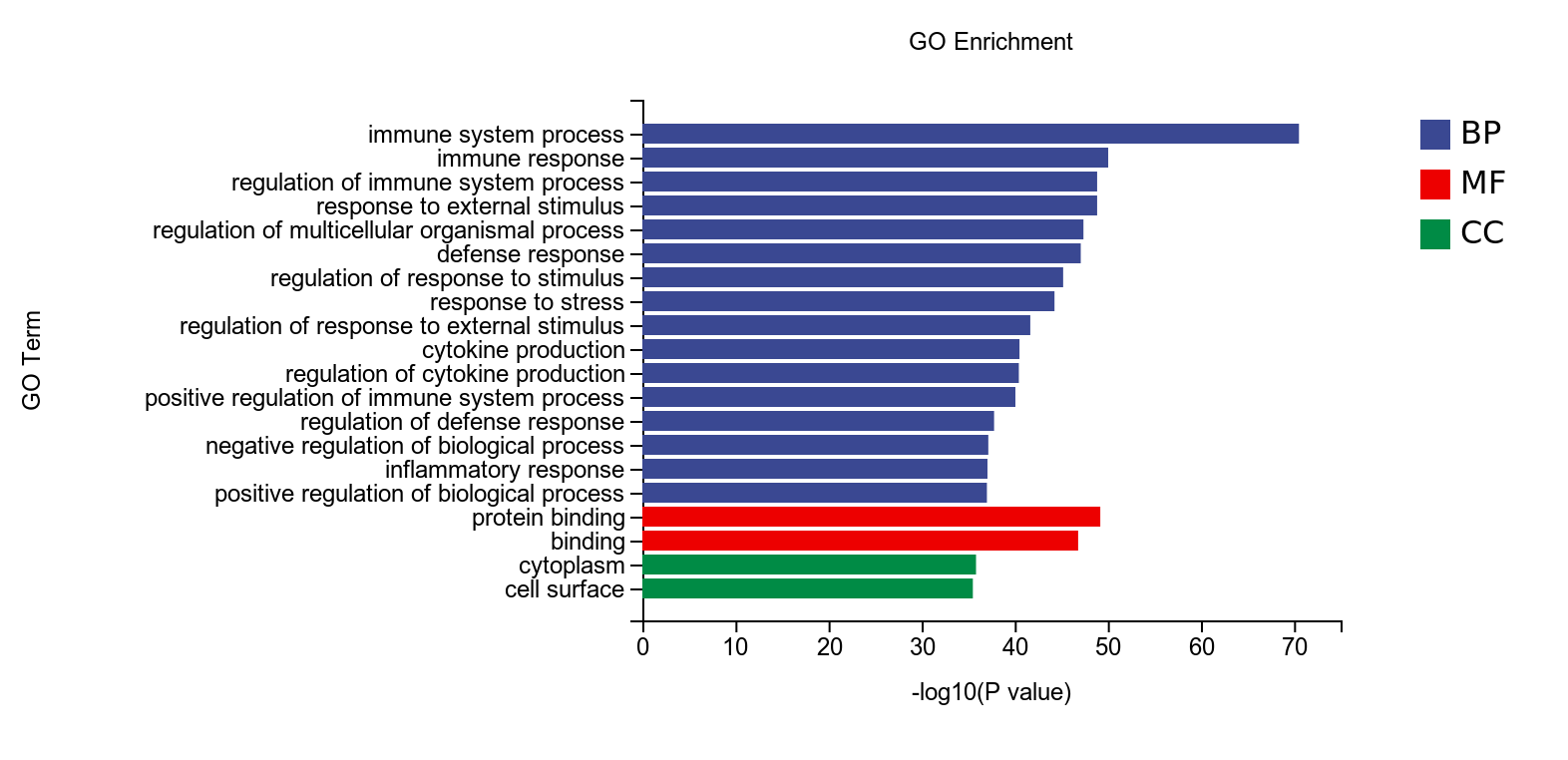


**Figure S23**. GO analysis of the identified differentially expressed genes.


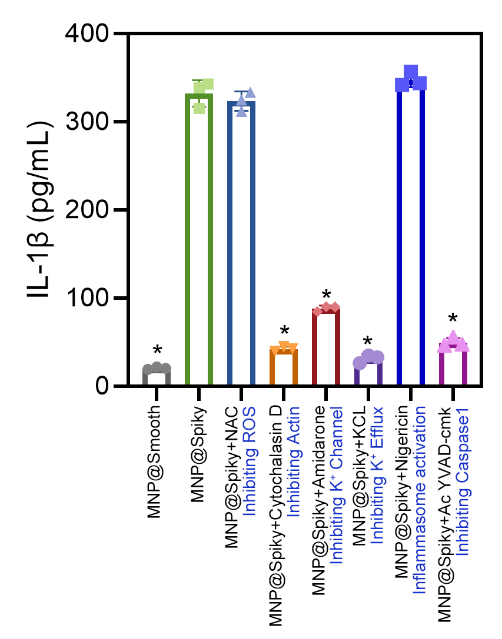


**Figure S24**. Exploration of the mechanism of DC activation by MNP@Spiky. The levels of IL-1β detected by ELISA after blocking different signaling pathways.


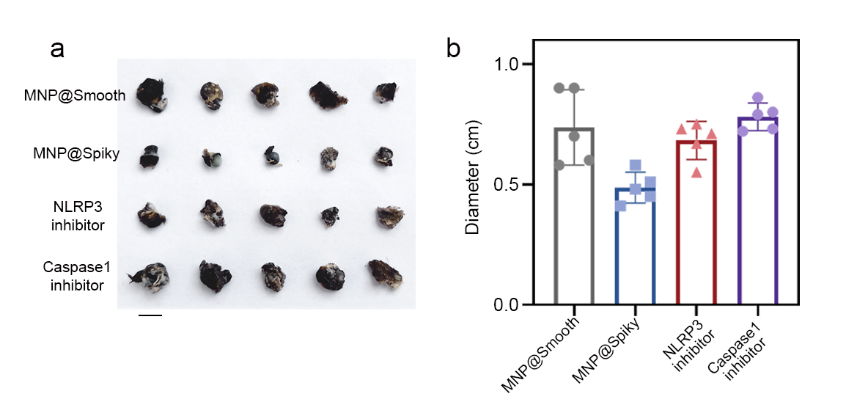


**Figure S25**. a) Photographs of dissected eyeballs in each group. Scale bar: 0.5 cm. b) Tumor volume represented by the diameter of eyeballs (n = 5).

**3. References**

1. X. Li, T. Yong, Z. Wei, N. Bie, X. Zhang, G. Zhan, J. Li, J. Qin, J. Yu, B. Zhang, L. Gan, X. Yang, *Nat Commun*. **2022**, 13, 2794.
